# *QuickStats*: Percentage* of Adults Aged ≥18 Years Who Lacked Reliable Transportation for Daily Living in the Past 12 Months,^†^ by Disability Status^§^ and Age Group — National Health Interview Survey, United States, 2022^¶^

**DOI:** 10.15585/mmwr.mm7307a4

**Published:** 2024-02-22

**Figure:**
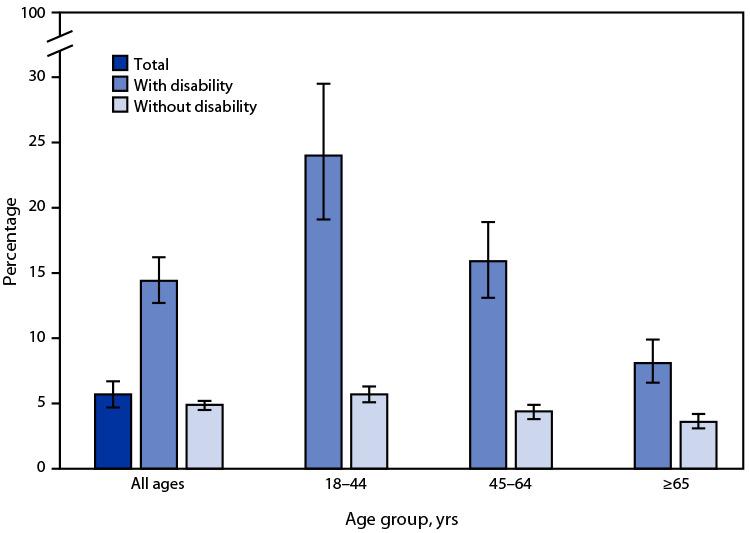
In 2022, 5.7% of adults aged ≥18 years lacked reliable transportation for daily living in the past 12 months. The percentage lacking reliable transportation for daily living among those with disability was higher (14.4%) compared with those without disability (4.9%). The percentages among persons with disability were higher than percentages among those without disability in all age groups (18–44 years: 24.0% versus 5.7%; 45–64 years: 15.9% versus 4.4%; and ≥65 years: 8.1% versus 3.6%). Regardless of disability status, the percentage of adults who lacked reliable transportation for daily living decreased with increasing age.

Source: National Center for Health Statistics, National Health Interview Survey, 2022. https://www.cdc.gov/nchs/nhis/index.htm

